# Bora Downregulation Results in Radioresistance by Promoting Repair of Double Strand Breaks

**DOI:** 10.1371/journal.pone.0119208

**Published:** 2015-03-05

**Authors:** Junmei Cairns, Yi Peng, Vivien C. Yee, Zhenkun Lou, Liewei Wang

**Affiliations:** 1 Department of Molecular Pharmacology and Experimental Therapeutics, Mayo Clinic, Rochester, Minnesota, 55905, United States of America; 2 Department of Biochemistry, Case Western Reserve University, Cleveland, Ohio, 44106, United States of America; 3 Department of Oncology and Oncology Research, Mayo Clinic, Rochester, Minnesota, 55905, United States of America; The University of Hong Kong, HONG KONG

## Abstract

Following DNA double-strand breaks cells activate several DNA-damage response protein kinases, which then trigger histone H2AX phosphorylation and the accumulation of proteins such as MDC1, p53-binding protein 1, and breast cancer gene 1 at the damage site to promote DNA double-strand breaks repair. We identified a novel biomarker, Bora (previously called C13orf34), that is associated with radiosensitivity. In the current study, we set out to investigate how Bora might be involved in response to irradiation. We found a novel function of Bora in DNA damage repair response. Bora down-regulation increased colony formation in cells exposed to irradiation. This increased resistance to irradiation in Bora-deficient cells is likely due to a faster rate of double-strand breaks repair. After irradiation, Bora-knockdown cells displayed increased G2-M cell cycle arrest and increased Chk2 phosphorylation. Furthermore, Bora specifically interacted with the tandem breast cancer gene 1 C-terminal domain of MDC1 in a phosphorylation dependent manner, and overexpression of Bora could abolish irradiation induced MDC1 foci formation. In summary, Bora may play a significant role in radiosensitivity through the regulation of MDC1 and DNA repair.

## INTRODUCTION

In response to DNA damage, cells activate the DNA damage response (DDR) that comprises initial sensing of DNA breaks, followed by downstream events leading to cell cycle arrest, DNA damage repair, and subsequent cell cycle resumption. A class of PI3K protein kinases, ATM, ATR and DNA-PK are the apical kinases of the DDR [1–4]. These kinases phosphorylate several proteins including histone H2AX, Chk1 and Chk2. Phosphorylation of H2AX at serine 139 promotes the assembly of DNA repair complexes at the damaged sites [5–6], while phosphorylation of Chk1 and Chk2 kinases activates these kinases, which in turn activate downstream effectors to induce cell cycle arrest and promote DNA repair [7–10]. If the damage cannot be repaired, it will lead to permanent growth arrest or apoptosis [11]. Numerous factors are involved in DNA double-strand breaks (DSB) signaling in response to irradiation (IR). Those factors accumulate at damaged sites in focal structures called IR-induced foci (IRIF). Specifically, γ-H2AX is bound through the tandem breast cancer gene 1 (BRCA1) C-terminal domain (BRCT) and domains of the DDR-mediator protein MDC1 [12–13]. MDC1 is phosphorylated by ATM, which then recruits the ubiquitin E3-ligase, RNF8, together with RNF168 to ubiquitylate histones H2A and H2AX and that, in turn, promotes accumulation of p53-binding protein 1 (53BP1) and BRCA1 [14–18].

We recently identified a novel biomarker for radiation response, Bora (C13orf34), by using a Genome-Wide Association Study (GWAS) performed with a panel of 300 human lymphoblastoid cell lines (LCLs) [19]. A correlation analysis between basal expression array data and radiation cytotoxicity in these LCLs identified Bora as one of the top candidates associated with radiation cytotoxicity [19]. As a cell cycle protein, Bora enhances the initial activation of Polo-like kinase 1 (PLK1) in an Aurora A-dependent manner during G2/M transition, and as a result facilitates G2/M transition [20]. However, how Bora regulates radiosensitivity remains unclear.

In the present study, we show that Bora contributes to radioresistance through direct involvement in the activation of the DNA damage checkpoint response and an increased rate of DNA repair. Bora-depleted tumor cells preferentially activate the DNA damage checkpoint in response to IR, and they repair damaged DNA more effectively than Bora-sufficient tumor cells. Mechanistically, we found that this sensitization is due to the inhibition of MDC1 and 53BP1 accumulation at the damage-repair site through direct binding of Bora to MDC1, leading to inhibition of the recruitment of other factors to the damage sites and, as a result, deficiency in DNA repair.

## MATERIALS AND METHODS

### Cell lines

Human pancreatic cancer HupT3 cell line were gifts from Dr. Daniel D. Billadeau, Mayo Clinic (ATCC, Manassas, VA,). Human cervical cancer Hela cell line and HEK 293T cells were obtained from the ATCC. A HeLa clone with the integrated HR reporter DR‐GFP was a gift from Dr. Maria Jasin (Memorial Sloan Kettering). Hela cells were cultured in DMEM medium containing 10% FBS. HupT3 and 293T cells were maintained in RPMI 1640 medium with 10% FBS. Hela DR-GFP cells were grown in DMEM medium supplemented with 700 ng/mL of puromycin in a humidified atmosphere with 5% carbon dioxide.

### Antibodies

Anti–phospho-Histone γ-H2AX (Ser139) was from Millipore (Billerica, MA); MDC1 and 53BP1 antibodies were gifts from Dr. Zhenkun Lou, Mayo Clinic. Anti-Bora was obtained from New England Peptide (Gardner, MA). Anti–HA, GST, anti-PLK1 as well as anti-pCDK9 and CDK9 were from Cell Signaling Technology, Inc (Danvers, MA); anti–FLAG and actin antibodies were purchased from Sigma-Aldrich, Inc. (St. Louis, MO); and the horseradish peroxidase–conjugated secondary antibodies against mouse and rabbit were obtained from Santa Cruz Biotechnology, Inc (Santa Cruz, CA). Fluorescent dye–conjugated secondary antibodies were obtained from Invitrogen Corp (Carlsbad, CA).

### Plasmids

pGEX-4T-1-MDC1 FHA, pGEX-4T-1-MDC1 BRCT, pIRES2-Bora, and pIRES2-Bora S501A mutant were gifts from Dr. Zhenkun Lou, Mayo Clinic. Bora N-terminus (1–312 aa) and Bora C-terminus (313–559 aa) were PCR amplified from the full length of Bora and cloned into pIRES2 vector (Dr. Zhenkun Lou, Mayo Clinic). Bora S325A mutant and S325E mutant was generated by the QuikChange Site-Directed Mutagenesis Kit (Stratagene, Santa Clara, CA).

### Cell culture and transfection

Bora siRNAs, PLK1 siRNA, Aurora A siRNA and negative control siRNA were purchased from QIAGEN (Valencia, CA). A second Bora siRNA was purchased from Dharmacon. Cdks 1, 2, 4, 5, 7, and 9 siRNAs were from Dharmacon (Lafayette, CO). Transfection of plasmids and siRNAs was performed with Lipofectamine 2000 and lipofectamine RNAiMAX reagents (Invitrogen, Carlsbad, CA), respectively. Bora shRNA was purchased from Origene (Rockville, MD). Bora stably knockdown cells were generated by transfecting HupT3 and Hela cell lines with lentiviral-delivered RNA interference. Briefly, adherent cells were treated with 0.5 mL of the virus followed by overnight incubation (37°C, 5% CO2). The next day, viral medium was replaced with fresh medium containing puromycin (1 μg/mL) to select a population of resistant cells.

### Cytotoxicity and clonogenic survival assays

Irradiation was performed using ^137^Cesium gamma-rays (J. L. Shepherd and Associates Mark I Model 25 Irradiator, San Fernando, CA). Cell viability was determined using the 3-(4,5-dimetilthiazol-2-yl)-5-(3-carboxymethoxyphenyl)-2-(4-sulfophenyl)-2H-tetrazolium, inner salt (MTS) assay according to the instructions of the manufacturer (Promega, Madison, WI). Specifically, 100 μl of cells (5x10^4^ cells/ml) were plated into 96-well plates, and were treated with ionizing radiation at 0, 0.25, 0.5, 1, 2.5, 5, 10, 20 and 40 Gy in triplicate at each radiation dose. After incubation for 3 days, the MTS assay was performed.

Cells were transfected with specific siRNA or plasmids for 24 h and 500 cells were plated in triplicate in 6-well plates. Cells were then treated with increasing doses of IR (0, 0.25, 0.5, 1, 2.5, and 5 Gy). After 7 or 14 d of incubation, the colonies were fixed with ice-cold methanol and stained with 0.05% crystal violet. Colonies containing >50 cells were counted.

### Real-time Quantitative Reverse Transcription-PCR

Total RNA was isolated from cultured cells with the Qiagen RNeasy kit (QIAGEN Inc. Valencia, CA), followed by QRT-PCR performed with the 1-step, Brilliant SYBR Green QRT-PCR master mix kit (Stratagene, La Jolla, CA). Specifically, primers purchased from QIAGEN were used to perform QRT-PCR using the Stratagene Mx3005PTM Real-Time PCR detection system (Stratagene, La Jolla, CA). All experiments were performed in triplicate with GAPDH as an internal control. Control reactions lacked RNA template.

### Cell cycle assay

Cells were plated in 60-mm dishes, transfected with negative siRNA or Bora siRNA, and were harvested by trypsinization after 48 h. After fixing, cells were stained with propidium iodide, and analyzed by a flow cytometer (BD FACScans). Three independent experiments were performed and at least 20,000 cells were counted, the proportions of cells in different cell cycle phases were gated and calculated using the software Flowjo 8.7.1(Tree Star, Inc.).

### Immunoprecipitation and immunoblotting

Cells harvested in PBS were lysed in RIPA buffer (10 mM Tris pH 8, 1% Triton-X-100, 0.1% deoxycholate, 0.1% SDS, and 150 mM NaCl) supplemented with protease and phosphatase inhibitors. Lysates were clarified by centrifugation (13,000 r.p.m., 20 min, 4°C) and 500 μg–1mg proteins were used per immunoprecipitation. Proteins were captured with the appropriate antibody and protein S-sepharose Fast-Flow (Sigma). Immunoprecipitation with rabbit serum or from cells that do not express epitope-tagged protein were used as negative controls. Proteins were resolved by SDS–PAGE, transferred onto nitrocellulose (Protran) and probed using the appropriate primary and secondary antibodies coupled to horse-radish peroxidase. Protein detection was performed with ECL reagents (GE Healthcare).

### Immunostaining procedure

To visualize IRIF, cells cultured on coverslips were treated with 10 Gy IR followed by recovery for 1 h. Cells were then washed with PBS, incubated in 4% paraformaldehyde for 10 min, and permeabilized in 0.5% Triton X-100 in PBS for 10 min, and blocked with 5% bovine serum albumin for 1 h at room temperature. Cells were then incubated with the primary antibody, anti-γ-H2AX (Ser139; 1:1,000), and anti-53BP1 (1:1,500) for 2 h. Samples were washed and incubated with secondary antibody for 60 min. Cells were then mounted in a vectashield mounting medium containing 4′, 6-diamidino-2-phenylindole (DAPI). γ-H2AX, MDC1 and 53BP1 foci were examined using a fluorescence microscope (CRG Precision Electronics).

### Dephosphorylation of Bora and GST-BRCT pull-down assay

Bora were collected and extracted with 0.1% NP-40, then added to lambda phosphatase reaction buffer containing 14.8 U/μl of lambda phosphatase (New England BioLabs) and protease inhibitors and incubated at 30°C for 20 min. MDC1 BRCT and FHA domains were expressed as GST fusions in *E*. *coli* and purified on glutathione Sepharose (Amersham Pharmacia Biotech). The total reaction volume was transferred to a tube containing prepared Glutathione Sepharose 4B beads, and the GST-BRCT pull-down analysis was performed.

### HR assay

To evaluate HR repair of DNA-DSBs, Hela cells containing the DR-GFP construct were transfected with the I-SceI expression vector. Transient expression of I-SceI endonuclease generates a DSB at the integrated green fluorescent protein (GFP) gene sequences and stimulates HR. 24 h later, the cells were transfected with Aurora A siRNA, Bora siRNA, or various Bora expression plasmids. GFP signal was assayed 3 days post-transfection on a FACSCalibre flow cytometer (BD, Biosciences).

### *In Vitro* kinase assay

*In vitro* kinase assay was performed with bacterially produced full-length Bora protein or Bora S325A mutant. Briefly, extracts (10 μg protein) were incubated in an initial reaction volume of 20 μl containing 10 μl diluted active CDK7/CyclinH1/MNAT1 or CDK9/CyclinK (SignalChem). A blank control was set up by replacing the substrate with an equal volume of distilled H_2_O. The reaction was initiated by the addition of 5 μl [^32^p]-ATP assay cocktail. The mixture was then incubated in a water bath at 30°C for 15 min. Phosphorylated Bora was visualized by autoradiography. Western blot was performed with anti Bora antibody to confirm the protein.

### Statistical analysis

All error terms are expressed as the standard error of mean. Significance levels for comparison of differences between groups in the *in vitro* experiments were analyzed by the Student’s t-test. The differences were considered significant when p-value was <0.05. All reported p-values are two-sided.

## RESULTS

### Decreased Bora expression enhances radioresistance

**Fig 1:**
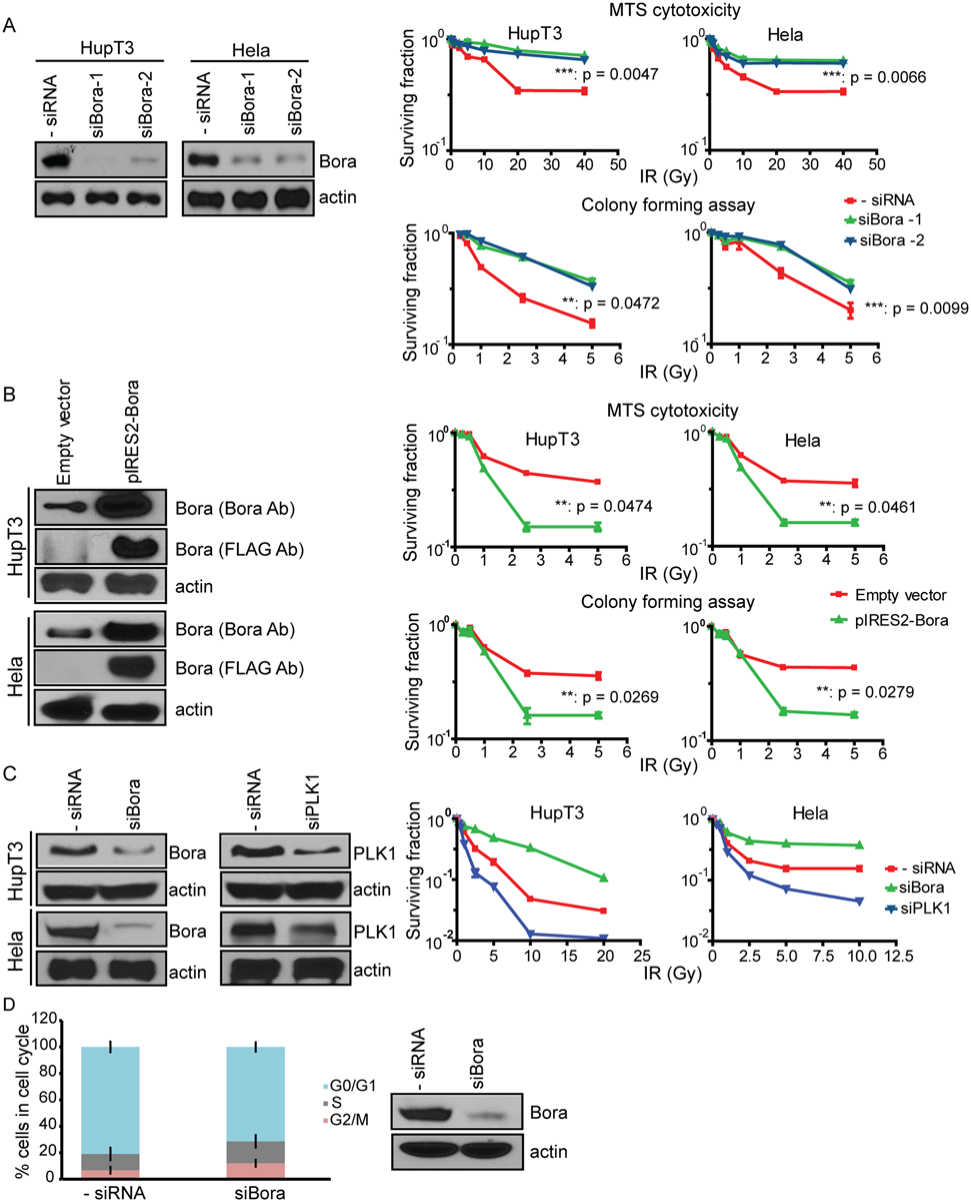
Effect of Bora on radiosensitivity and cell cycle in human tumor cell lines. A. Downregulation of Bora desensitizes cells to IR. Knockdown in HupT3 and Hela cells was performed using two Bora specific siRNAs, followed by MTS assay (upper panel) and colongenic assays (lower panel) to determine cell survival after treatment with increasing doses of IR. ** indicates p<0.05. Western blot was performed to determine the knockdown efficiency (left panel). B. Overexpression of Bora in HupT3 and Hela cells sensitizes cells to IR treatment as determined by MTS assay (upper panel), and clonogenic assays (lower panel). Western blot was performed to determine the overexpression efficiency (left panel). All experiments were performed in triplicate and error bars represent SEM of three independent experiments. Significance was defined by comparing the AUC values of radiation cytotoxicity curves between control and Bora knockdown cells. The x-axis indicates the radiation dose, and the y-axis indicates the surviving fraction after radiation exposure. ** indicates p<0.05. C. Bora affects radiosensitivity independent of its role in PLK1 pathway. HupT3 and Hela cells were transfected with indicated siRNAs, followed by MTS assay with increasing dosage of IR. Western blot was performed to determine the knockdown efficiency (left panel). All experiments were performed in triplicate and error bars represent SEM of three independent experiments. D. Down regulation of Bora slightly increased cells in S and G/M. HupT3 cells transfected with negative or Bora siRNAs were subjected to flow cytometry. Proportion of cells in each cell cycle was quantified. Western blot was performed to determine the knockdown efficiency (left panel). Error bar represents 3 independent experiments.

To determine the role of Bora in IR response, endogenous Bora was knocked down using siRNA in human pancreatic HupT3 and cervical Hela cancer cell lines, followed by exposure to increasing doses of IR (Fig. 1A). Knockdown of Bora desensitized both HupT3 and Hela cells to radiation treatment (Fig. 1A). Similar reductions in IR sensitivity were observed with a second siRNA, indicating that these results are not due to off-target effects of the siRNA. To further confirm the effect of Bora on radioresistance, we overexpressed Bora and found that ectopic expression of Bora increased radiosensitivity (Fig. 1B). Taken together, these results indicate that the down regulation of Bora significantly enhances IR resistance in both cells tested. Next we wanted to determine the mechanisms by which Bora might affect radiosensitivity. Bora is a known cofactor of Aurora-A, and together, they facilitate the phosphorylation of PLK1 at Thr-210 and influence the regulation of mitosis [21]. Therefore, we next determined whether Bora regulates IR response is related to the PLK1 pathway. Specifically, we performed knockdown of PLK1 and Bora and then determined the response to IR treatment. Interestingly, knockdown PLK1 had opposite effect on radiosensitivity (Fig. 1C). Since Bora is a cell cycle protein, to determine whether the effect on DNA repair is a secondary effect from cell cycle regulation, we knocked down Bora and measured cell cycle profiles. We found that downregulation of Bora only slightly increased S and G/M population (Fig. 1D). These results suggest that Bora regulates IR response through a mechanism that is independent of its role in PLK1 and cell cycle.

### Down regulation of Bora increases IR-induced DNA repair rate

**Fig 2:**
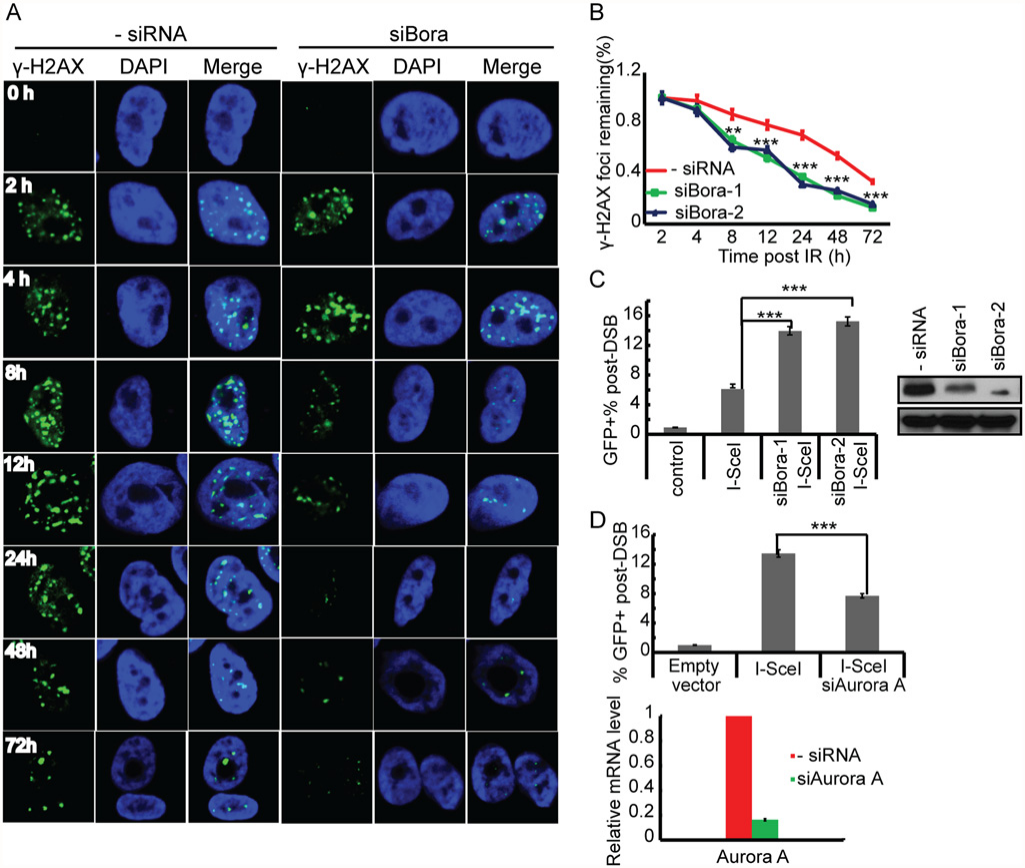
Effect of Bora on DNA repair. A. Bora knockdown results in a faster repair of DSBs. HupT3 cells were transfected with negative or Bora siRNA and treated with 10 Gy IR for indicated period of time, followed by fixation and staining with anti-γH2AX antibody and DAPI at each time point. B. Quantification of the γH2AX foci in Bora knockdown and control HupT3 cells at different time points post IR (error bars indicate SEM.; n = 100 cells per time point per experiment). The graph indicates the percentage of positive γH2AX foci at indicated time points after IR. There was a significant difference in the number of γH2AX foci between Bora knockdown cells and control cells over time after IR treatment. C. Downregulation of Bora increases IR-induced DNA repair. Control or Bora knocked down Hela cells were transfected with an I-SceI expression vector. Flow cytometric analysis was used to determine the percentage of GFP positive cells as an indication of DNA repair capability of I-SceI-generated DSBs. The data are presented as mean ± SEM from three independent experiments. *** indicates p<0.01. Western blot was performed to determine knockdown efficiency. D. Downregulation of Aurora A decreases IR-induced DNA repair. Control or Aurora A knocked down Hela cells were transfected with an I-SceI expression vector. Percentage of GFP positive cells was determined by flow cytometric analysis. The data are presented as mean ± SEM from three independent experiments. *** indicates p<0.01. qRT-PCR was performed to determine knockdown efficiency.

Enhanced DSB repair is an important mechanism by which cells may become resistant to IR [22]. We next examined whether Bora might also affect DNA repair and cell recovery after DNA damage. To test this possibility, we measured γ-H2AX nuclear foci formation using immunofluorescence analysis, since γ-H2AX nuclear foci formation has been widely used as an indicator of the presence of damaged DNA [5]. HupT3 cells were transfected with negative siRNA or 2 Bora siRNAs, and were then treated with IR. Treated cells were allowed to recover for various time periods before staining with γ-H2AX antibody to assess nuclear foci formation after DNA damage. Twenty four h after IR, the fraction of cells containing γ-H2AX nuclear foci and the number of foci in each cell were significantly decreased in Bora siRNA transfected cells (Fig. 2A, ***p<0.01), indicating a significantly faster rate of DSB repair in Bora knockdown cells (Fig. 2B). For example, 78% repair was completed 48 h following IR treatment in Bora knockdown cells, compared with only 46% in control cells (Fig. 2B, ***p<0.01). These data indicated that the downregulation of Bora increased the rate of DNA repair. To confirm this result, we also examined the effect of Bora on homologous- repair (HR). We performed a gene conversion assay using a previously established Hela cell line with integrated direct repeat-GFPs (DR-GFPs) (a Hela clone carrying the DR–GFP homologous recombination reporter) [23]. Specifically, we transfected the Hela cells with an I-SceI expression vector together with negative siRNA or Bora-specific siRNA. Expression of the endonuclease I-SceI in the cells leads to double-strand breaks in the DR-GFP reporter construct, and these breaks can be repaired by HR, producing GFP-positive cells. Therefore, the efficiency of HR repair is measured by the intensity of GFP signals using flow cytometry. As a negative control, cells transfected with empty vector containing no I-SceI were analyzed in parallel. As shown in Fig. 2C, Bora depleted cells consistently showed a more than two fold increase in GFP signals in the presence of I-SceI when compared with negative siRNA transfected cells (p<0.01). In addition, knockdown of Aurora A kinase, a kinase for which Bora binds to and together, regulates cell cycle progression, decreased GFP signal, indicating reduced DNA repair (Fig. 2D). This result again, suggests that the effect of Bora on DNA repair is independent of its function in cell cycle regulation. Collectively, our results indicate that Bora might play an important role in IR-induced DNA damage and repair.

### Bora prevents accumulation of activated DNA repair proteins MDC1 and 53BP1 at sites of IRIF

**Fig 3:**
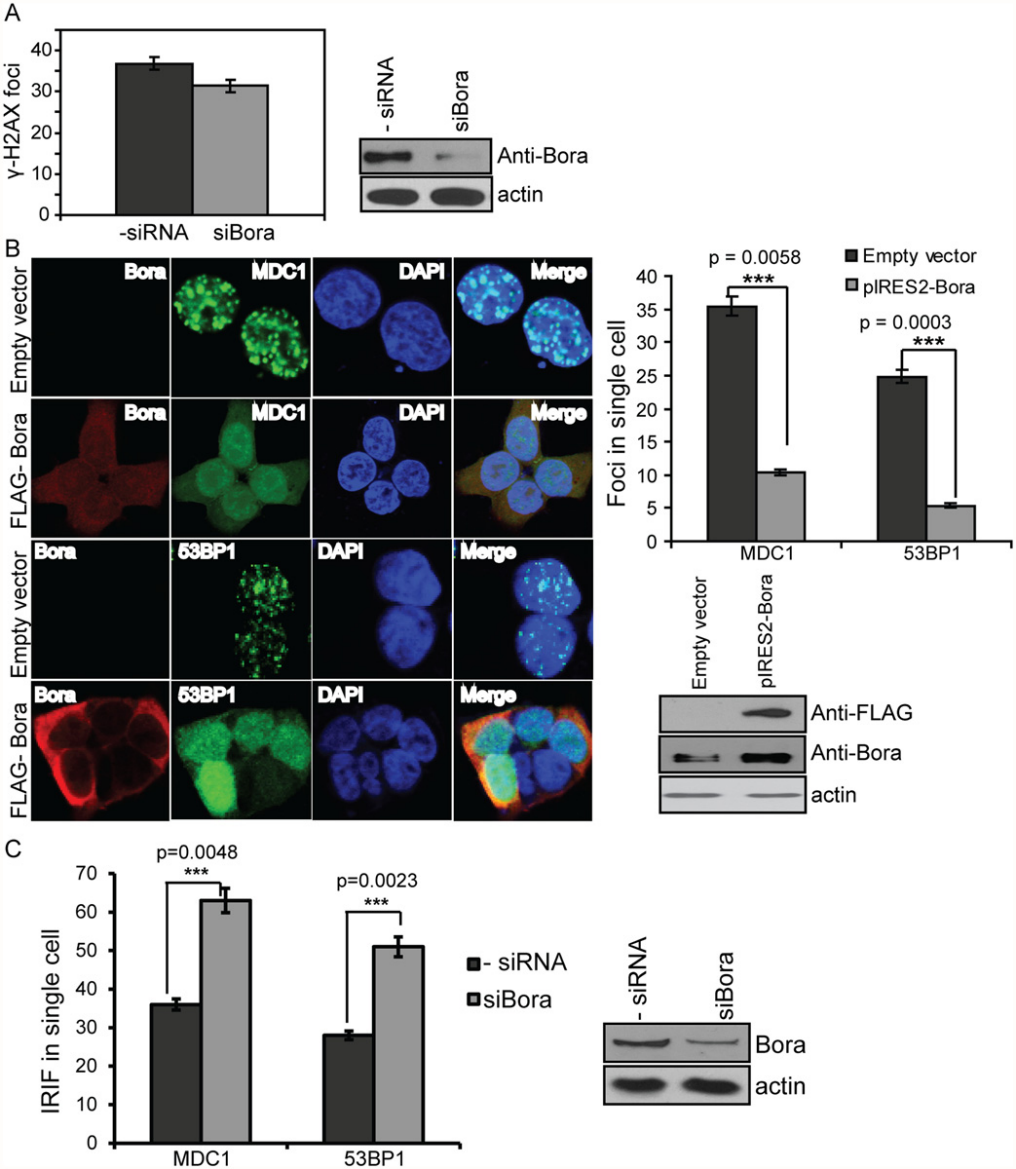
Bora prevents MDC1 and 53BP1 foci formation but not γH2AX. A. HupT3 cells were transfected with FLAG-tagged Bora, irradiated by 10 Gy and 0.5 h later stained with anti-FLAG antibody and γH2AX antibody, respectively. γH2AX foci were counted. At least 100 cells were counted in each experiment. Results are represented as number of foci per cell. Westernblot was performed to determine knockdown efficiency. B. MDC1 and 53BP1 foci formation in control HupT3 cells or cells with Bora overexpression. Cells were immunostained with anti- FLAG (red), MDC1 or 53BP1 (green) and DAPI (blue) antibodies. Quantification of MDC1 and 53BP1 foci. MDC1 and 53BP1 foci were counted and at least 100 cells were counted in each experiment. Results are represented as number of foci per cell. *** indicates p<0.01. Overexpression efficiency was indicated by Western blot analysis using anti-FLAG and anti-Bora antibody. C. Quantification of MDC1and 53BP1foci formation in control HupT3 cells or cells with Bora knockdown. Cells were immunostained with anti- FLAG (red), MDC1 or 53BP1 (green) and DAPI (blue) antibodies. Quantification of MDC1 and 53BP1 foci was performed in a similar fashion as described in Fig. 3B, and *** indicates p<0.01. Knockdown efficiency was determined by Western blot analysis using anti-Bora antibody.

To further examine how Bora might affect IR-induced DNA damage and repair, we investigated whether Bora regulates the recruitment of repair mediator proteins such as MDC1 and 53BP1to the DNA damage foci. Recruitment of these repair proteins to sites of DNA DSBs serves to amplify DNA damage signaling and to facilitate repair [24]. This process can be monitored by immunofluorescence for the rapid formation of distinct IR-induced foci (IRIFs). We overexpressed Bora in HupT3 cells, and 48 h after transfection, the cells were treated with 10 Gy IR, and immunostaining with antibodies against γ-H2AX, MDC1, and 53BP1 was performed. Overexpression of Bora alone was not sufficient to inhibit γ-H2AX foci (Fig. 3A), so initial phosphorylation of H2AX in response to IR was not significantly affected. However, overexpression of Bora resulted in a significant reduction of IRIF formation of MDC1 and 53BP1 (Fig. 3B), while downregulation of Bora increased IRIF formation (Fig. 3C). Our data suggested that Bora blocks the recruitment of downstream factor of γ-H2AX even though γ-H2AX foci itself was not affected.

### MDC1 binds to Bora and affects DNA damage repair response and radiosensitivity

**Fig 4:**
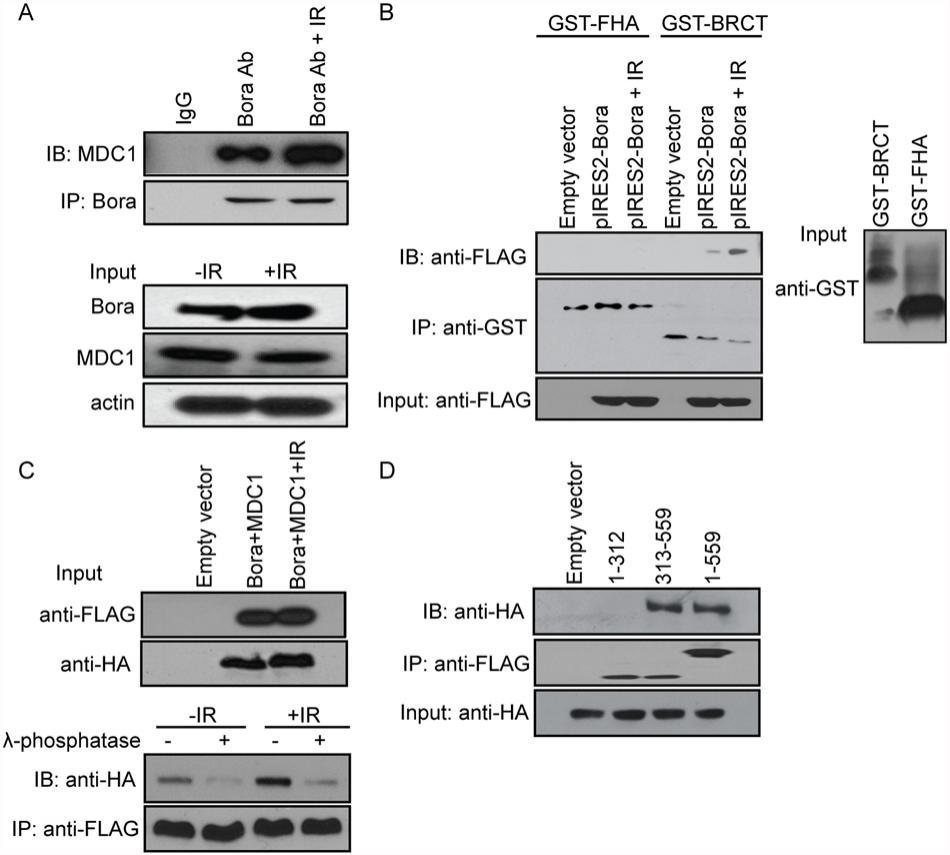
Bora inhibits MDC1 foci formation *via* interaction with MDC1 BRCT domain in a phosphorylation-dependent manner. A. Bora interacts with MDC1. IP was performed using anti-Bora antibody followed by blotting with anti-MDC1 antibody in 293T cells. B. Bora interacts with MDC1 *via* the MDC1 BRCT domain. Lysates from 293T cells overexpressing FLAG-tagged Bora were incubated with GST-BRCT or GST-FHA fusion protein immobilized on the glutathione agarose beads for 2 h before washing. The elution was subsequently analyzed by Western blot with anti-FLAG antibody. C. Effect of phosphorylation on Bora-MDC1 interaction. Lysates from 293T cells overexpressing FLAG-tagged Bora and HA-tagged MDC1 was either incubated with buffer alone or with lambda phosphatase for 15 min at 30°C. The mixture was then incubated with FLAG beads. There was a significant decrease in the binding between Bora and HA-tagged MDC1 in the presence of lambda phosphatase regardless of IR treatment. D. Bora C terminus fragment (313–559 aa), but not N terminus (1–312 aa) co-immunoprecipitates with HA-tagged MDC1. 293T cells were co-transfected with plasmids encoding FLAG-tagged Bora or various deletion constructs, and plasmids encoding HA-tagged MDC1. Lysates were incubated with FLAG beads, followed by Western blot analysis with anti-HA antibody.

**Fig 5:**
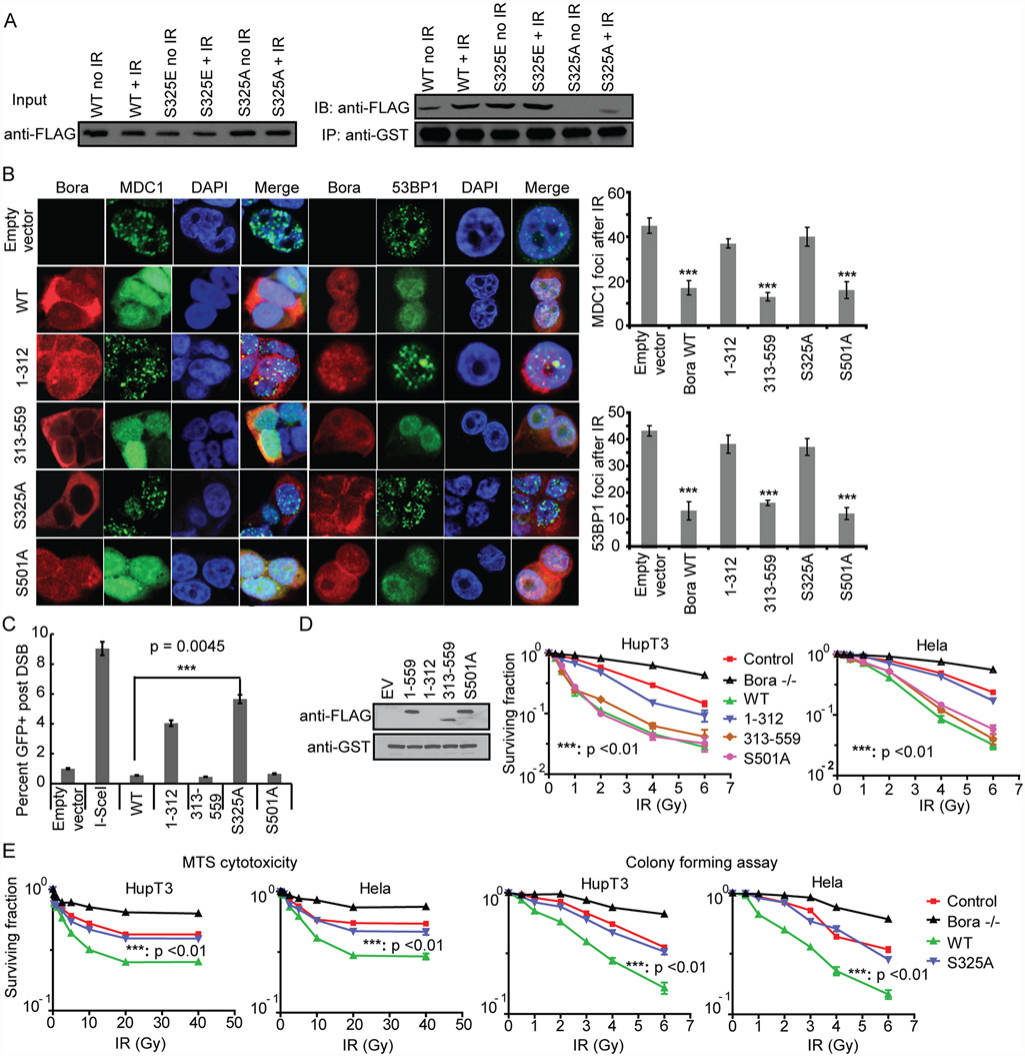
Bora S325A phosphorylation mutant causes increased MDC1 and 53BP1 IRIF formation, DNA repair and resistance to IR treatment. A. Bora S325 phosphorylation is required for its association with MDC1. Lysates from 293T cells overexpressing FLAG-tagged WT Bora, the S325E mutant and the S325A mutant with or without IR treatment were incubated with GST- MDC1 BRCT fusion protein immobilized on the glutathione agarose beads and subsequent analysis by Western blotting with anti-FLAG antibody. B. Effect of Bora deletion and mutant constructs on MDC1 and 53BP1 IRIF formation. Left Panel: Foci formation. Hela cells were transfected with wild type, Bora deletion, S501A or S325A mutant FLAG-tagged constructs. Forty-eight h after the transfection, cells were treated with 10 Gy IR and immunostained with indicated antibodies. Right Panel: Quantification of MDC1 and 53BP1-IRIF formation per cell is shown after 10 Gy IR. Error bars represent SEM calculated based on100 cells. C. Percentage of GFP positive cells observed in DR-GFP reporter assay in Hela cells that overexpressed different Bora mutant or deletion constructs. Data are presented as mean ± SEM from three independent experiments. Significance was calculated between WT Bora and S325A mutant. D. Effect of Bora deletion and S501mutant constructs on the Bora binding to MDC1 and IR sensitivity. Left Panel: Immunoprecipitation. Lysates from 293T cells overexpressing FLAG-tagged WT Bora and Bora mutants as well as Bora N or C terminal constructs were incubated with GST- MDC1 BRCT fusion protein immobilized on the glutathione agarose beads, with subsequent Western blot analysis with anti-FLAG antibody. Right Panel: Colony forming assays. Bora stably knockdown cell lines were transfected with WT Bora, S501A or C and N terminal constructs, and then treated with increasing dose of IR and cytotoxicity was determined by colony forming assays. E. Effect of S325 mutant construct on IR sensitivity. HupT3 and Hela cell lines with Bora stably knockdown were transfected with WT Bora and S325A mutant, and then treated with increasing dose of IR and cytotoxicity was determined by MTS assays and colony forming assays. P-values were calculated for the difference in AUC values between WT and S325A mutant.

Because MDC1 directly binds γ-H2AX, and MDC1 recruitment to the sites of DNA damage was blocked, it is likely that Bora affect the interaction between MDC1 and γ-H2AX. We next tested whether MDC1 interacts with Bora. As shown in Fig. 4A, MDC1 interacted with Bora, and the interaction increased after cells were treated with ionizing radiation. On the contrary, we did not observe interactions between 53BP1, Chk2, ATM and p53 and Bora (data not shown). MDC1 contains two phosphor-specific protein binding domains: an N-terminal FHA domain and a C terminal tandem BRCT domain. GST pull-down experiments performed with bacterially expressed GST fusions with either FHA or BRCT domains showed that only the tandem BRCT domains bound to Bora (Fig. 4B). Because BRCT domains recognize phosphor-Ser/Thr motifs [25], it is likely that MDC1 recognizes and binds to phosphorylated Bora following DNA damage. Indeed, the interaction between Bora and MDC1 was abolished in the presence of λ-phosphatase (Fig. 4C), indicating that Bora might bind to MDC1 in a phosphorylation dependent manner. To further study the interaction between Bora and MDC1, we mapped the regions of Bora that might interact with MDC1. Using truncated Bora mutants, we found that the region spanning residues AA 313 to 559 of Bora mediated the interaction between Bora and MDC1 BRCT domains (Fig. 4D). In order to map the phosphorylation sites of Bora, we carried out LC–MS/MS analysis of the Bora using immunoprecipitated lysates from the 293T cells overexpressing Bora. Phosphorylation analysis of Bora showed that S188, S252, and S325 were phosphorylated after DNA damage (Table 1). We choose S325 for further characterization since it is located within the region that bound to MDC1. To further confirm S325 is required for Bora-MDC1 interaction, we evaluated the binding of the Bora phospho-mimetic mutant (S325E) and the Bora phospho-deficient mutant (S325A) with MDC1. The binding between WT Bora and GST-MDC1 increased after IR treatment. The Bora phospho-mimetic mutant (S325E) also showed a strong binding with GST-MDC1 with or without IR treatment (Fig. 5A). Furthermore, the phospho-deficient mutant (S325A) abolished the MDC1–Bora interaction (Fig. 5A), confirming that the phosphorylation of S325 is required for MDC1 binding.

**Table 1:** Mass spectrometry analysis of the affinity purified Bora immuno-complex in Her293-T cells overexpressing flag tagged Bora.

3 confidently assigned phosphorylation sites
SPYIDGC**S**#PIK (325)
SPLQTPSSGQFSS**S**#PIQASAK (252)
ADEFADQSPGNL**S**#SSSLR (188)

We next investigated the effect of Bora deletion constructs and phosphorylation mutant constructs on IRIF formations and response to IR treatment. Specifically we tested Bora deletion or mutant constructs for their ability to prevent IRIF accumulation. We also included the S501A mutant, which was previously identified [26] and was in the Bora-MDC1 binding domain. However, we did not observe this site to be phosphorylated after IR treatment during our LC-MS/MS analysis.

The Bora C terminus fragment (313–559 aa), S501A mutant and WT Bora all prevented the accumulation of MDC1 and 53BP1 at IRIF to a similar degree (Fig. 5B). However, we observed that the N terminal domain and S325A, the mutant deficient in MDC1 binding, were unable to prevent IRIF formation (Fig. 5B). These data suggested that the Bora-MDC1 interaction is dependent on S325 phosphorylation and that this process is responsible for preventing the recruitment of MDC1 and 53BP1 to damage sites. We also measured the efficiency of HR in cells transfected with Bora mutants using the previously described DR-GFP reporter. The C terminal construct (313–559) fragment, the S501A mutant and WT Bora showed the same inhibition effects on HR following I-SceI cleavage. However, overexpression of Bora N-terminal fragment (1–312 aa) and S325A mutant resulted in significantly less inhibition of HR (55% and 37% decrease in GFP positive cells, respectively) (Fig. 5C), indicating a role for Bora in the regulation of HR after DSB. Thus, the interaction between the MDC1 BRCT domain and Bora might prevent MDC1 IRIF formation and blocks DNA repair.

Finally, we determined the effect of the MDC1-Bora interaction and the S325 phosphorylation site on response to IR. We found that the previously identified S501 did not affect Bora binding to MDC1 after IR induced DNA damage (Fig. 5D left panel). We then reconstituted the S501 mutant and Bora deletion constructs in cells depleted endogenous Bora by stably knockdown using Bora shRNA, and observed that the S501 mutant showed similar IR sensitization as did wild type Bora (Fig. 5D, p<0.01). Consistently, the Bora C terminus, a region that interacts with MDC1, showed similar sensitization to IR treatment as did wild type Bora and the S501 mutant (Fig. 5D, p<0.01). However, the Bora N terminal fragment failed to sensitize tumor cells to IR treatment (Fig. 5D), indicating that the interaction between Bora and MDC1 is necessary for the radiosensitization effect of Bora. Finally, overexpression of the phospho-deficient mutant S325A Bora construct in cells depleted endogenous Bora was not able to sensitize cells to IR treatment, indicating that S325 was required to confer the radiosensitivity effect of Bora in both HupT3 and Hela cells (Fig. 5E, p<0.01).

### Cyclin-dependent kinase (CDK)-dependent phosphorylation of Ser325 is required for the Bora-MDC1 interaction

**Fig 6:**
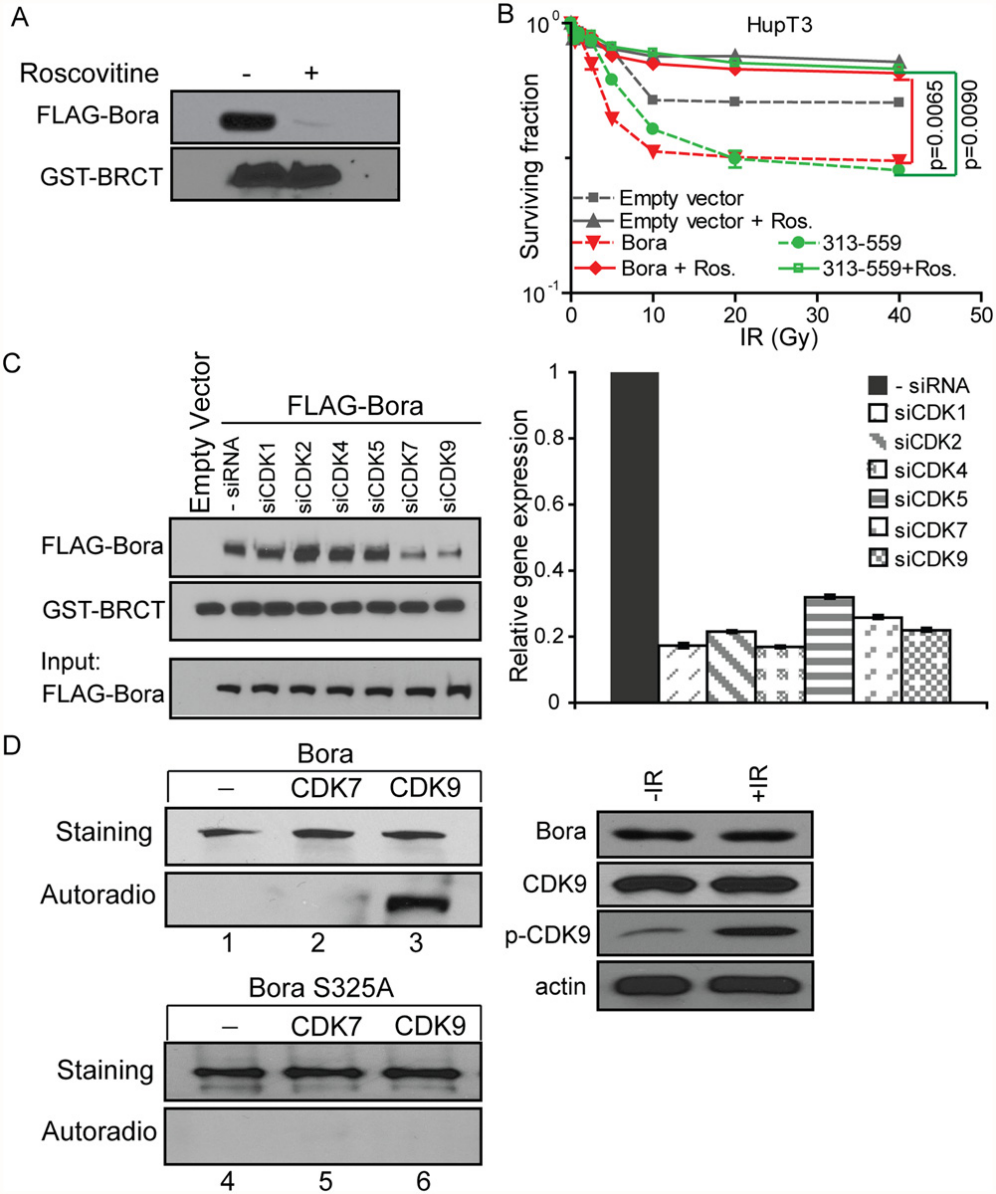
Bora Ser325 can be phosphorylated by CDK9. A. CDK inhibitor blocks the interaction between MDC1-BRCT domain and Bora. FLAG-Bora from 293T cells was incubated *in vitro* with bacterially purified GST-BRCT domain of MDC1 in the presence of roscovitine. Interaction was determined by Western blot analysis. B. Roscovitine causes resistance to IR treatment in HupT3 and Hela cell lines overexpressing Bora. IR+20 μM roscovitine resulted in resistance phenotype when compared with treatment by IR alone in Bora overexpressed cells. All experiments were done in triplicate, and error bars represent SEM of three independent experiments. C. Depletion of CDK7 and CDK9 by RNAi impairs the association between Bora and MDC1. 293T cells overexpressing Bora were transfected with siRNA targeting different CDK family members. Binding assays were performed by incubating purified FLAG–Bora with bacterial purified GST–MDC1-BRCT fragment. qRT-PCR was performed to determine knockdown efficiency. D. *In vitro* phosphorylation assay shows that Bora is phosphorylated by CDK9, and CDK9 is activated upon IR treatment. Left panel: Bacterial purified full-length Bora or Bora S325A mutant was incubated in the presence of [γ-^32^P] ATP with either CDK7/CyclinH1/MNAT1 or CDK9/CyclinK as indicated. Bora phosphorylation was determined by autoradiography. Bora protein levels were determined by Coomassie blue-staining. Right Panel: HupT3 cells were treated with 10 Gy IR and blotted with indicated antibodies. Data are representative of three independent experiments.

Finally, in order to identify the kinase(s) that might phosphorylate Bora S325, we examined the sequence around S325. We noticed the presence of a perfect consensus CDK phosphorylation site sequence around S325 (S/T*-P-x-K/R, where x = any amino acid and the asterisk indicates the site of phosphorylation) [27]. The interaction between FLAG-Bora and the GST- MDC1 BRCT domain was then determined in the absence or presence of roscovitine, a CDK inhibitor. Addition of roscovitine for 24 h reduced the interaction dramatically (Fig. 6A). To determine whether CDKs might be involved in regulation of the IR response in Bora over-expressed cells, we studied the effect of CDK inhibition by roscovitine on radiosensitivity. Pretreatment of HupT3 cells transfected with full length Bora or the C terminus of Bora (313–559) with 20 μM roscovitine significantly decreased radiosensitivity (Fig. 6B). We acknowledge that roscovitine can block cells in G1 and that might affect survival independent of regulation of Bora phosphorylation. Therefore, we also performed knockdown studies of individual CDK to further validate our hypothesis—phosphorylation of Bora plays an important role in its interaction with MDC1, and thus response to IR treatment. In order to further narrow down which CDK family members might be responsible for the phosphorylation of Bora S325 [28], we knocked down individual CDKs (1, 2, 4, 5, 7, and 9) to determine their effect on the Bora-MDC1 interaction. As shown in Fig. 6C, the interaction between Bora and MDC1 was reduced significantly after knockdown of CDK7 or CDK9. We then performed *in vitro* kinase assays and found that CDK9/CyclinK, but not CDK7/CyclinH1/MNAT1, was able to phosphorylate Bora (Fig. 6D, lanes 1 to 3). However, Bora S325A, the mutated form that lack of the Ser325 site, was not phosphorylated (lanes 4 to 6). To test whether IR might activate CDK9, we performed western analysis and found that treatment of IR increased phosphorylated CDK9 (Fig. 6D).

## DISCUSSION

Bora is the first evolutionarily conserved activator of Aurora-A [21]. Aurora-A plays an important role in centrosome maturation, spindle assembly, and asymmetric protein localization during mitosis [29]. Because Aurora A and PLK1 play important roles in promoting cell cycle transition under normal conditions or during DNA damage recovery, it is essential to downregulate the activity of CDKs, PLK1, and Aurora A in cells experiencing DNA damage to maintain the G2 checkpoint and allow time for accurate DNA repair. It has been reported that BRCA1 binds to PLK1 and concomitantly inhibits the kinase activity of PLK1 [30]. BRCA1 likely participates in the early inhibition of PLK1 activity as well as the subsequent inhibition observed during the period of DNA damage-induced cell cycle arrest [30]. In our study, we found that Bora inhibits MDC1 and 53BP1 recruited to the IRIF after IR treatment, but not BRCA1 (data not shown), and we also found that PLK1 knockdown had opposite effect on radiosensitivity comparing with Bora knockdown, indicating that Bora affects IR response through a different pathway than PLK1 (Fig. 1C). Knocking down Bora only slightly increased the S and G/M cells, indicating that the phenomena we observed of Bora on IR response and DNA damage and repair might not be a secondary effect resulted from changes in cell cycle (Fig. 1D). In addition, MDC1 foci formation is also not cell cycle dependent. Knocking down Aurora A showed opposite effect on DNA repair e.g. reduced DNA repair, as compared with down regulation of Bora (Fig. 2D). Taken together, our results indicate that Bora might be involved in the regulation DNA damage and repair pathway independent of its role in cell cycle.

DNA damage checkpoint pathways maintain genomic integrity by regulating DNA repair, cell cycle progression and apoptosis [31]. The ATM pathway mainly responds to DNA DSBs induced by various insults including IR, but also to other types of DNA damage [32–33]. Histone H2AX phosphorylation on serine 139 by ATM or by other DNA damage response kinases on a C-terminal serine residue to form γ-H2AX is a critical early event in the chromatin response to DNA double strand breaks in eukaryotes [34–35]. γ-H2AX, in turn, recruits the chromatin-associated adaptor protein, MDC1 [26], which recruits 53BP1 through RNF8/RNF168 [16]. Our results showed that IR induced formation of γ-H2AX to IRIF was not affected by Bora level (Fig. 3), but rather that there was significantly more rapid kinetics of γ-H2AX foci formation, indicating faster DSB repair by homologous repair in Bora downregulated cells (Fig. 2). These observations suggest that Bora may not be an initial sensor of genomic damage but rather functions as a downstream mediator or modulator of DNA damage signals. Once IR induced DSBs occur, the DNA damage site recruits a series of proteins to these sites to start DNA repair; included among these proteins are MDC1 and 53BP1. 53BP1 mediates IR-induced ATM S1981 autophosphorylation. MDC1 and 53BP1 collaborate to promote IR-induced Chk2 T68 phosphorylation and to mediate pATMS1981, pChk2T68 and NBS1 foci formation [36]. We have shown that Bora can bind to MDC1 and inhibit MDC1 recruitment to IRIF after DNA damage (Figs. 3 and 4). Bora overexpression blocked the recruitment of MDC1 to nuclear foci (IRIF) after IR treatment (Fig. 3). The effect of Bora on the recruitment of MDC1 and 53BP1 to IRIF after DSB-induced damage suggests that Bora may play a direct role in DNA damage responses.

Bora Interacts with Aurora A, and the interaction is abrogated by deleting the Bora N-terminal region (Δ1–66) [37]. We found that Bora C terminus was responsible for binding and regulating MDC1 recruitment to the damage site. In addition, we also showed that the phosphorylation of Bora Ser325 is critical for MDC1 interaction (Figs. 4 and 5). Future studies will be needed to determine characteristics of Bora binding partners. One possibility might relate to different phosphorylation sites on Bora. A recent study reported that GSK3β activity is required for Bora-mediated mitotic entry as a result of S274 and S278 phosphorylation [38]. We also showed that CDK9 was responsible for the phosphorylation of Bora S325 and, in turn, regulate the binding between Bora and MDC1 (Fig. 6). Future studies will be also needed to investigate whether additional kinases might be involved in the phosphorylation of Bora that contributes to the interaction between Bora and MDC1. We also found that, although CDK7 knockdown inhibits the binding between MDC1 and Bora, we did not observe *in vitro* phosphorylation by CDK7. CDK7 is a potential activator of CDK9. CDK9 full activation might require T-loop phosphorylation by CDK7 [39]. Therefore, even though CDK7 did not directly phosphorylate Bora, it might be involved in the process through activation of CDK9.

Finally, our observation added another layer of regulation of DNA damage and repair pathway. We know that DNA repair is tightly regulated. For example, hyperactivation of non-homologous end joining (NHEJ) could result in chromosome translocation. Recent studies also suggest that Rap80, a BRCA1 binding protein that colocalizes with BRCA1 at the sites of DNA damage, inhibits BRCA1’s function in homologous recombination (HR) [40]. Therefore, DNA repair need to be fine-tuned. Our study is the first characterization of Bora function in DNA repair. The cellular context by which Bora functions in DNA repair needs to be further characterized.

In summary, in this study we have provided evidence showing that Bora is associated with a protein complex containing MDC1, and is involved in the regulation of DNA damage repair. Downregulation of Bora increased DNA repair induced by IR. Therefore, mutations in the Bora gene that result in aberrant expression or protein function might have an effect on genome integrity and response to IR. The insights that we obtained could help to individualize cancer therapy by selecting the most appropriate therapy and by predicting response phenotypes.
